# Scale, trust, and the digital divide: a systematic review of AI and ML for agricultural applications

**DOI:** 10.3389/frai.2026.1798896

**Published:** 2026-06-19

**Authors:** Richa Bhattarai, Jennifer Koch, Wolfgang Jentner, Mehreen Habib, Michael C. Wimberly, David Ebert

**Affiliations:** 1 Department of Geography and Environmental Sustainability, The University of Oklahoma, Norman, OK, United States; 2 Data Institute for Societal Challenges, The University of Oklahoma, Norman, OK, United States; 3 School of Civil Engineering and Environmental Science, The University of Oklahoma, Norman, OK, United States; 4 Laboratory of Geo-information Science and Remote Sensing, Wageningen University & Research, Wageningen, Netherlands; 5 The University of Arizona, Tucson, AZ, United States

**Keywords:** agricultural production, digital divide, farming operations, review, *Zero Hunger*

## Abstract

Artificial Intelligence (AI) and Machine Learning (ML) have transformed the agricultural sector and will continue to do so. Adopting AI/ML technology has the potential to help meet global food, feed, and fiber demands while promoting sustainable farming practices. However, adoption depends on multiple dimensions of trust, particularly trust in system performance and trust in data governance. In this systematic review, we analyze scientific literature for the current status of trust in AI/ML technology for agricultural applications. We use the PRISMA protocol to identify 38 peer-reviewed publications and analyze the content along three dimensions: farming operation scale, trust in AI/ML technology, and the digital divide. Across literature, low trust (66%) and a pronounced digital divide (66%) are dominant themes. Five interconnected clusters, namely data governance, lack of technical skills, lack of explainability, lack of reliability, and high cost of technology, emerge as major barriers shaping trust and adoption. Despite these barriers, farmers across all scales express interest in AI/ML when tools clearly reduce risk or improve yields. However, trust is influenced not only by transparency, but also by structural factors such as data governance, power asymmetries, and unequal access to digital infrastructure. Transparency regarding algorithms' functionality, especially regarding the use of sensitive data, will be important for addressing a lack of trust in the new technology. Training and educating farmers in the use of new tools and technologies, especially at small and medium-scale farming operations, can reduce the digital divide. Targeting intermediate-scale farming operations may further support more inclusive adoption pathways. These improvements could help shift the geographic focus of AI/ML applications in agriculture, supporting progress toward the *Zero Hunger* Sustainable Development Goal.

## 1 Introduction

Artificial intelligence (AI) has made significant contributions to transforming the agricultural sector and traditional farming practices. The use of AI in agriculture can be traced back to the introduction of advanced computing capabilities and the growing availability of data during the 1980s (Bacco et al., 2019; Bannerjee et al., 2018). Over time, AI algorithms and machine learning (ML) techniques have been developed and tailored specifically for agricultural applications. With the increased availability of sensors, drones, satellites, and other data-gathering technologies, farmers have obtained access to a wealth of data on crops, environmental conditions, and field conditions. This vast amount of information creates an opportunity for AI/ML applications, which excel in processing and analyzing large datasets (Javaid et al., 2023). AI/ML has been used to improve efficiency through crop management, pest and disease management, soil and irrigation management, weed management, yield predictions, and cost savings (Bannerjee et al., 2018; Ben Ayed and Hanana, 2021). A widespread adoption of AI in farming practices has the potential to help the agricultural sector meet the global challenge of producing more food while conserving resources and promoting sustainable agricultural practices, and work toward accomplishing the *Zero Hunger* Sustainable Development Goal (Klerkx et al., 2019).

According to consulting company Precedence Research (Precedence Research Report, 2023), the global market size for AI in agriculture (which includes the development, sale, and implementation of AI-based tools, software, and technologies) was assessed to be USD 1.37 billion in 2022, and it is expected to exceed approximately USD 11.13 billion by 2032, showing a Compound Annual Growth Rate (CAGR) of 23.3% from 2023 to 2032. As AI/ML applications are expected to continue to shape the future of agriculture, it is important to make them safe, reliable, and trustworthy. The capacity of these applications to establish and nurture trust among farmers plays a vital role in the adoption and successful implementation of AI technologies in the long run (Kaur et al., 2023).

The European Union proposed seven key requirements to make AI trustworthy, namely (1) human agency and oversight, (2) technical robustness and safety, (3) privacy and data governance, (4) transparency, (5) diversity and fairness, (6) societal well-being, and (7) accountability (Smuha, 2019). Meeting these requirements is a prerequisite for ensuring that AI/ML applications are used responsibly and ethically in the agricultural sector. Recent research on agricultural AI and agri-food ethics argues that generic trustworthy AI frameworks risk overlooking sector-specific power asymmetries, data commodification, and structural injustices, and calls for governance approaches that are sensitive to farm scale (Ryan et al., 2023; Van Hilten et al., 2025; Hirunyatrakul, 2025).

Beyond governance frameworks, trust in AI/ML applications is also shaped by how end users perceive the reliability, usability, and transparency of these systems in practice (Thiebes et al., 2021; Glikson and Woolley, 2020). Trust in AI/ML applications is often connected to the explainability of AI and its application outcomes—a topic addressed in the domain of explainable AI (XAI) (McDermid et al., 2021). XAI focuses on developing explainable techniques that make AI processes and the resulting predictions and decisions transparent, document the strengths and weaknesses of the process, and allow users to verify and validate AI-driven recommendations (Rai, 2020). In the agricultural context, XAI aims to enable farmers to identify and correct potential errors and ensure the accuracy of AI applications. This transparency enhances understanding of AI's decision-making and thereby cultivates confidence of farmers in the reliability of AI applications (Ryo, 2022). The reliability of AI applications means that AI systems can deliver accurate and viable results over time, even in novel situations and under changing environmental conditions (McDermid et al., 2021).

Ethical considerations for AI in agriculture involve the careful examination of the moral implications and responsibilities associated with the development, deployment, and use of AI in farming practices (Dara et al., 2022). Additionally, the ethical implications of AI on labor practices, socioeconomic disparities, and environmental impacts are significant and require careful consideration (Tai, 2020). This means thinking not only about what AI can do, but also about who benefits, who might be left behind, and what new risks might be created. It also requires ensuring that farmers have sufficient information and control to make informed decisions about using AI, such as understanding how their data will be used, whether the technology will change their workload or job roles, and whether the environmental impacts of AI-enabled farming align with long-term sustainability goals (Van Hilten et al., 2025; Jakku et al., 2019).

Distinctive challenges facing the agricultural sector include changing weather patterns, local variability in soil and other environmental factors, and pest and disease outbreaks. Difficult to predict weather patterns, including extreme events such as droughts and floods, create challenges for farmers to plan effectively, impacting both short-term decision-making and long-term strategies (Jain et al., 2015). Soil properties such as moisture levels, pH levels, nutrient and organic matter content, and environmental factors, including water availability and topography, can vary significantly even at a field scale (Cambardella et al., 1994). The emergence of pests and diseases in regions where they were previously unheard of presents new problems for farmers (Chakraborty and Newton, 2011). To address these challenges, AI systems need to be adaptable, capable of mitigating risk, acknowledge tradition, support long-term relationships, secure data privacy, and have the capabilities to enhance human collaboration (Hossen et al., 2023). These considerations emphasize that tools and technologies must be sufficiently reliable to justify building trust in AI.

Given the characteristics of the agricultural sector, certain aspects of these challenges are more important for building trust compared to trustworthy AI in general, and therefore, it is important to prioritize the characteristics for the widespread adoption of AI technologies in the agriculture sector. Several recent reviews of AI in agriculture have been published, showing a general increase in research on this topic. Existing publications usually focus on topics such as AI methods and techniques used, benefits of smart farming, challenges of technology costs, and the lack of skill. For instance, Hossen et al. (2023) present information about the transformative impact of AI on agriculture, including crop monitoring, soil management, weather forecasting, irrigation, weeding, disease and pest management, and the use of drones and robots, discussing challenges like technology costs and farmer training. Elbasi et al. (2023) present the benefits and challenges of AI applications and compare and discuss several AI methodologies used in smart farming, including machine learning, expert systems, and image processing. They identify challenges such as data collection and accuracy, environmental sustainability, security issues, and a lack of skills and knowledge. Oliveira and Silva (2023) present the benefits, challenges, and trends of AI in agriculture and identify machine learning, convolutional neural networks, Internet of Things (IoT), big data, robotics, and computer vision as the most used AI technologies in agriculture. They recognize challenges, including resource optimization, integrating AI technologies into existing agricultural processes, data management, affordability, labor qualification, and supportive public policies.

This systematic review explores scientific literature discussing challenges and strategies for improving trust in AI in the agricultural sector and for farmers' decision-making. By doing so, it aims to systematically address the challenges of building trust among farmers for the adoption of AI technologies in the agricultural sector, especially based on the organizational scale of farms (i.e., the operational characteristics of a farm based on farm size and management approach) and the associated digital divide. While only a few prior studies (Gardezi et al., 2023; Sullivan et al., 2024) specifically address concerns related to building trust in AI for agricultural applications, no existing review has examined how trust varies across farm organizational scales and how these differences intersect with the digital divide. Addressing this gap, we cover the recent literature spanning the years 2018 to August 2025 and organize our findings along three axes: (1) the scale of farming operations, (2) trust in AI and ML, and (3) the perceived digital divide. By doing so, we aim to provide a synthesis of scientific literature on how trustworthy AI can be implemented in agriculture to enhance sustainable and resilient farming practices and discuss challenges and opportunities.

## 2 Materials and methods

### 2.1 Manuscript selection

**Figure 1:**
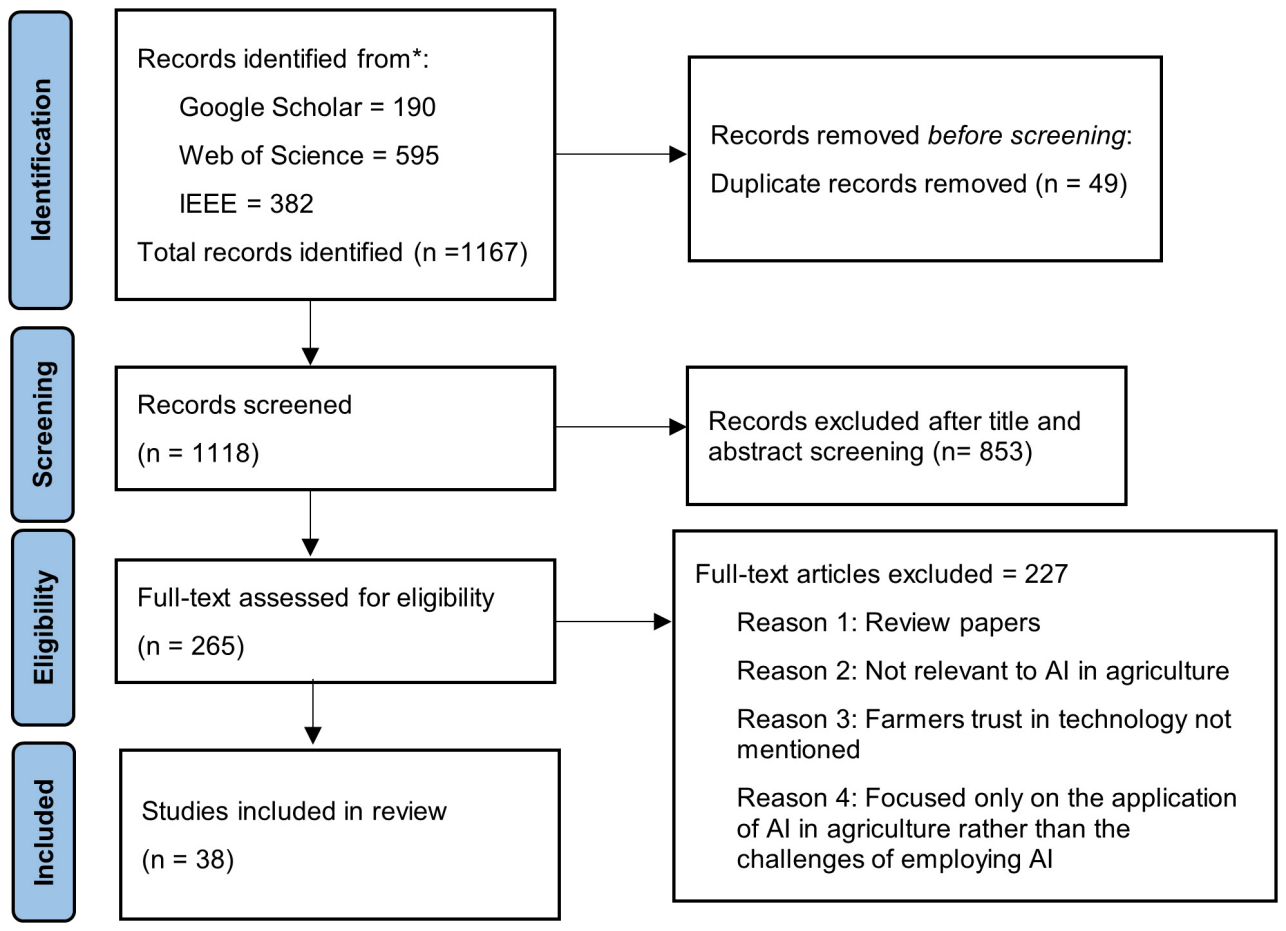
PRISMA flow diagram outlining the protocol adopted for this systematic review, including the number of articles at each stage, based on (Siddaway et al., 2019).

AI applications provide many advantages to the agricultural sector, like improved decision-making, increased efficiency, and financial gain, while decreasing environmental impacts (Jakku et al., 2019). However, barriers to adoption due to a lack of farmers' trust in AI technologies still exist (Alexander et al., 2024). To identify these challenges and strategies for improving the trustworthiness of AI technology applications for the agricultural sector, a systematic review of the scientific literature was conducted, following the PRISMA (Preferred Reporting Items for Systematic Reviews and Meta-Analyses) protocol described by Siddaway et al. (2019). This protocol includes the stages (1) Identification, (2) Screening, and (3) Eligibility evaluation and study quality to guide the selection of articles (4) Included for systematic review. Figure 1 shows the flow of the systematic review and reports the number of manuscripts filtered at each stage for this study.

#### 2.1.1 Identification

Advanced searches were performed in Google Scholar, Web of Science, and IEEE Xplore to identify relevant studies using the following keywords. The syntax was adjusted to match the respective database search requirements and is provided in Supplementary material S1.

(“AI” OR “artificial intelligence” OR “machine learning” OR “data science”) AND (“agri^*^” OR “farm^*^” OR “digital agriculture” OR “precision agriculture”) AND (“trustworthy” OR “trustable” OR “transparent” OR “reliable” OR “trust” OR “explainable” OR “XAI” OR “interpretable“ OR “ethical”)

The search terms were selected based on our research topic's keywords and their frequently used synonyms. The term “remote sensing” was excluded to avoid over-representation of highly technical image-processing studies lacking discussion of trust or adoption challenges. In addition, review articles (including systematic reviews and meta-analyses) were excluded from the search to ensure the synthesis focused on primary empirical research that directly investigates, measures and reports trust in agricultural applications. While this approach enables consistent analysis of observed practices and experiences, it may limit inclusion of broader conceptual perspectives on trust, power asymmetries, and governance. Using these search queries in the listed databases and limiting our search to papers written only in English and published from 2018 to August 2025, yielded 1,167 publications. Removing duplicate manuscripts reduced the number of publications from 1,167 to 1,118.

#### 2.1.2 Screening

In the next step, the titles and abstracts of the 1,118 articles were screened. Articles that were irrelevant to AI in agriculture or focused solely on the application of AI in fields other than agriculture were excluded. One hundred and ninety seven papers from the title screening and 656 from the abstract screening were removed, for a total of 853. This reduced the number of publications from 1,118 to 265. Most excluded articles focused on AI but only mentioned agriculture as one domain where AI is flourishing.

#### 2.1.3 Evaluation of eligibility and study quality

The remaining 265 articles were then assessed for eligibility. To decide whether to include or exclude publications from the corpus for our review, we read the full text and excluded articles not presenting original research (i.e., review papers); articles not relevant to AI technology in the agriculture sector; articles not mentioning topics related to farmers' trust in AI technology; and articles focused only on the application of AI in agriculture rather than the challenges of employing AI in the agriculture sector. This evaluation step removed an additional 227 publications, leaving 38 manuscripts.

#### 2.1.4 Included manuscripts

Screening and eligibility assessment in accordance with the PRISMA protocol identified 38 studies for inclusion in the systematic review. The information on the selected publications, including title, authors, publication year, country, journal name, and publication databases, is provided in Supplementary material S2.

### 2.2 Quality assessment

To evaluate the methodological robustness of the reviewed literature, the first author conducted a structured quality assessment using a predefined scoring protocol. Each selected study was evaluated using three criteria: methodological robustness, data source reliability, and empirical grounding. Each criterion was scored on a scale from 0 to 2, where 0 indicates the criterion is not addressed, 1 indicates partial or implicit consideration, and 2 indicates the criterion is clearly addressed. Methodological robustness was assessed based on the clarity of the study design, the appropriateness of the methods, and the transparency of the analysis. Data source reliability was evaluated based on the credibility, origin, and adequacy of the data. Empirical grounding examined whether the findings were supported by real-world data, experiments, or validated case studies. Based on the total score, studies were categorized as high quality (5–6), medium quality (3–4), or low quality (0–2) (Supplementary material S2). To ensure compliance with systematic review reporting standards, a completed PRISMA 2020 checklist is appended (Supplementary material S3).

### 2.3 Analysis framework

Various challenges and recommendations to improve the trustworthiness of AI technologies in agriculture were identified by analyzing the full texts of the 38 articles identified (Supplementary material S2). To communicate the results in an effective and reproducible manner, our findings are organized along three dimensions: (1) the organizational scale of the farming operation, (2) trust in AI and ML technology, and (3) the perceived digital divide. For each dimension, operational definitions were developed prior to information extraction to guide consistent classification.

#### 2.3.1 Organizational scale

Based on the organizational scale of the farming operation, three broad categories are used: small-scale farms, large-scale farms, and an intermediate scale, i.e., a farming operation scale that is neither small nor large. The included papers originate from multiple countries, and none of the selected papers for review mention a specific criterion or a specific size cut-off for this scale categorization. Instead, they describe these categories qualitatively, referring to size and management approach. Farms were classified according to how the authors described operational size, ownership structure, and management style. Therefore, for this paper, the implied meanings of these categories are used. Small-scale farms are those that cover smaller areas, produce smaller quantities of agricultural products, are often traditional, individually or family-operated, and frequently qualify as organic. Many of these farms have limited resources and often rely on traditional and organic farming methods to grow a variety of crops. Large-scale farms include big agricultural operations and industrial-scale farms and are often owned by corporations. These farms typically have substantial resources to further increase their efficiency and yield and focus more on monocultures of high-value and cash crops. Intermediate-scale farms are those that cannot be categorized as either small- or large-scale farms. Thus, scale classification was context-sensitive rather than globally standardized, reflecting the relative position of farms within each country's agricultural structure. For our analysis purposes, small-scale farms are referred to as low, intermediate-scale farms as medium, and large-scale farms as high organizational scale.

#### 2.3.2 Trust in AI/ML technology

Based on the level of trust in AI and ML, farms are categorized into three broad categories: AI-hesitant, AI-adoptive, and farms with an intermediate level of trust. This classification is used as an analytical operationalization of trust in technology adoption, reflecting different degrees of confidence in the performance, reliability, and governance of AI/ML systems reported in the reviewed articles. AI-hesitant farms include farms that are skeptical about using AI technologies. AI-adoptive farms include farms that actively adopt AI/ML technologies and have relatively higher expectations for their performance and usefulness. Intermediate farms are neither hesitant nor overly eager to consider AI. For our analysis, AI-hesitant farms are referred to as low-trust farms, intermediate farms as medium, and AI-adopting farms as high-trust farms.

#### 2.3.3 Digital divide

Digital divide refers to the gap between the technological and skill requirements needed to effectively use AI/ML tools in agriculture and the actual access, infrastructure, and digital skills currently available to farmers. This includes disparities in internet connectivity, access to digital devices and platforms, digital literacy, data skills, and experience with advanced technology (Royall, 2021). Based on the perception of the digital divide described in the manuscripts, papers are categorized into three categories: low, medium, and high. A low digital divide means that the farmers possess both the technological access and skills required to engage with AI/ML tools. In contrast, a high digital divide means that the required technologies and competencies exceed what is currently available to most farmers, creating a larger gap between AI system demands and farmers' capacities. A medium digital divide reflects a situation where access and skills are improving but remain insufficient to fully support AI/ML adoption.

A “no information provided” category or “NA” was also included for all three axes.

### 2.4 Coding

Information on the farm's organizational scale, farmers' trust in AI and ML technologies, and the digital divide from the selected papers were extracted and recorded in the respective categories by the first author using the predefined operational definitions (Table 1). The coding was conducted in two rounds. After the initial classification, all studies were reviewed again to ensure that the assigned categories were consistent with the operational criteria. When any case was unclear, the study was re-examined and compared with others before a final decision was made.

**Table 1:** List of manuscripts included in the analysis and their categories along the three dimensions.

Author(s)	Year	Organizational scale of farm				Trust in AI/ML				Digital divide			
		Low	Medium	High	NA	Low	Medium	High	NA	Low	Medium	High	NA
Fleming et al.	2018	X				X						X	
Eastwood et al.	2018		X			X					X		
Dharmaraj et al.	2018	X							X			X	
Jakku et al.	2019		X			X						X	
Wiseman et al.	2019	X	X	X		X						X	
Wolanin et al.	2020		X	X					X				X
Tsakiridis et al.	2020	X	X	X		X							X
Orn et al.	2020	X				X						X	
Sparrow et al.	2021	X	X	X		X						X	
Gardezi and Stock	2021	X	X	X				X				X	
Heldreth et al.	2021	X				X						X	
Camaréna et al.	2021	X	X				X					X	
Durrant et al.	2021		X			X					X		
Ryo	2022			X		X							X
Raturi et al.	2022	X	X	X		X					X		
Sabrina et al.	2022				X				X				X
Gardezi et al.	2022	X	X	X		X						X	
Cartolano et al.	2022				X	X							X
Alexander et al.	2023	X	X	X		X						X	
Hüllmann et al.	2023	X				X						X	
Dilleen et al.	2023	X		X		X	X	X				X	
Adereti et al.	2023	X	X	X		X						X	
Ryan et al.	2023	X	X	X		X						X	
Marinko et al.	2023	X	X	X		X	X					X	
Czibere et al.	2023		X	X			X				X		
Mallinger et al.	2024	X	X	X		X						X	
Rozenstein et al.	2024	X	X	X		X							X
Vardhan et al.	2024				X				X				X
Bampasidou et al.	2024				X	X						X	
Mabkhot et al.	2024	X	X				X					X	
Deji et al.	2024		X	X			X					X	
Gent	2025	X					X						X
Tahir et al.	2025	X							X			X	
Yang et al.	2025				X		X						X
Hirunyatrakul et al.	2025	X					X					X	
Van Hilten et al.	2025	X	X	X		X						X	
Ryan et al.	2025	X	X	X		X						X	
Kramarz et al.	2025	X				X						X	

This classification along three dimensions enables us to do a cross-axis analysis to understand how these three factors interact and influence the potential for AI use in agriculture. Based on this analysis, challenges specific to the organizational scale of farming operations, along with factors influencing farmers' hesitancy or adoption of AI/ML technology and the influence of the digital divide are identified. Potential reasons why farmers may want to adopt AI in their operations are also described.

## 3 Results

### 3.1 General overview of included papers

**Figure 2:**
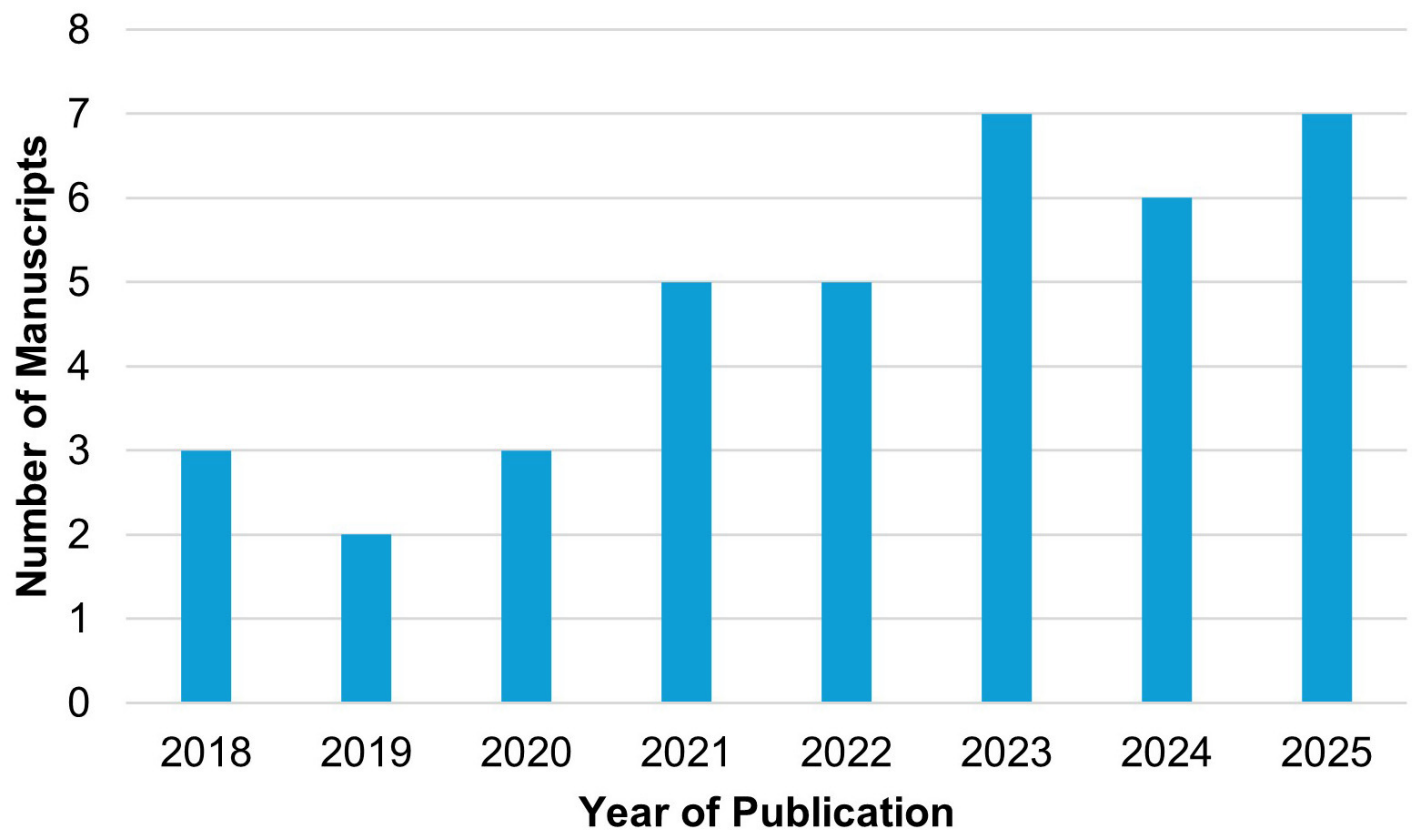
Distribution of selected manuscripts by year of publication.

We analyze 38 papers published between 2018 and August 2025. The quality assessment indicates that the majority of studies demonstrate high methodological robustness, with 32 studies classified as high quality, 5 as medium quality, and 1 as low quality. The summary of the reviewed studies is included in Supplementary material S2. Figure 2 displays the number of papers published per year. We conducted the literature review in late fall of 2025. Hence, the 2025 bar in the figure shows an incomplete number of papers for that year. While the publication numbers are low for 2018–2020, they increase from 2023 to 2025. We expect the number of papers on trust in AI/ML in agriculture to continue growing.

**Figure 3:**
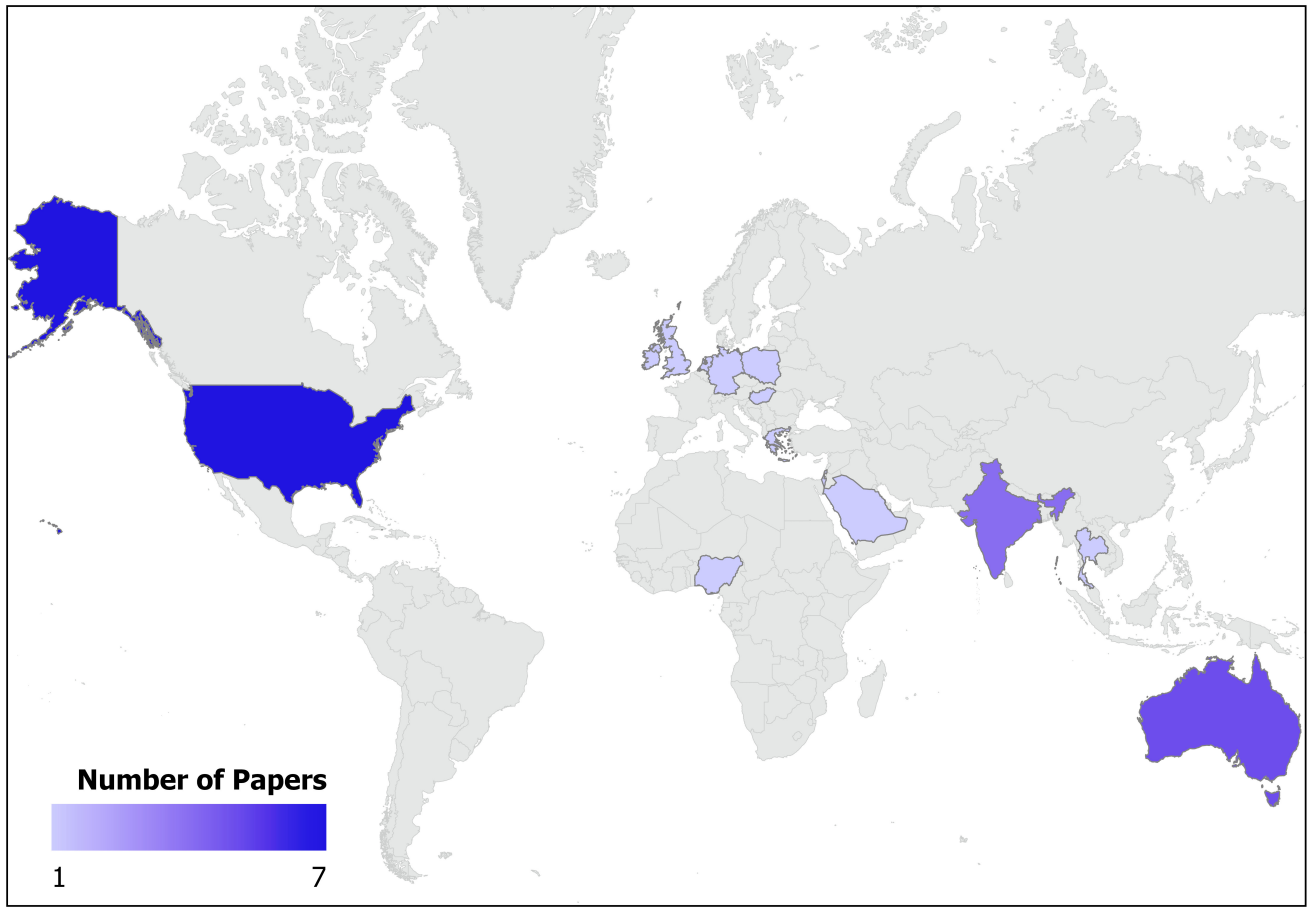
Geographic distribution of the reviewed studies.

Most of the 38 reviewed studies were conducted in high-income countries, with the largest numbers from the United States (7 papers) and Australia (5 papers), followed by 9 papers from European countries, including Germany, Greece, Hungary, Ireland, Israel, the Netherlands, Poland, and the UK (Figure 3). Four papers focus on India. A small number of studies focus on other countries, including Nigeria (1 paper), Thailand (1 paper), and broader regional categories such as the Global South (2 papers), Africa (1 paper). Four papers adopt a global perspective, whereas three do not mention any geographic context.

**Figure 4:**
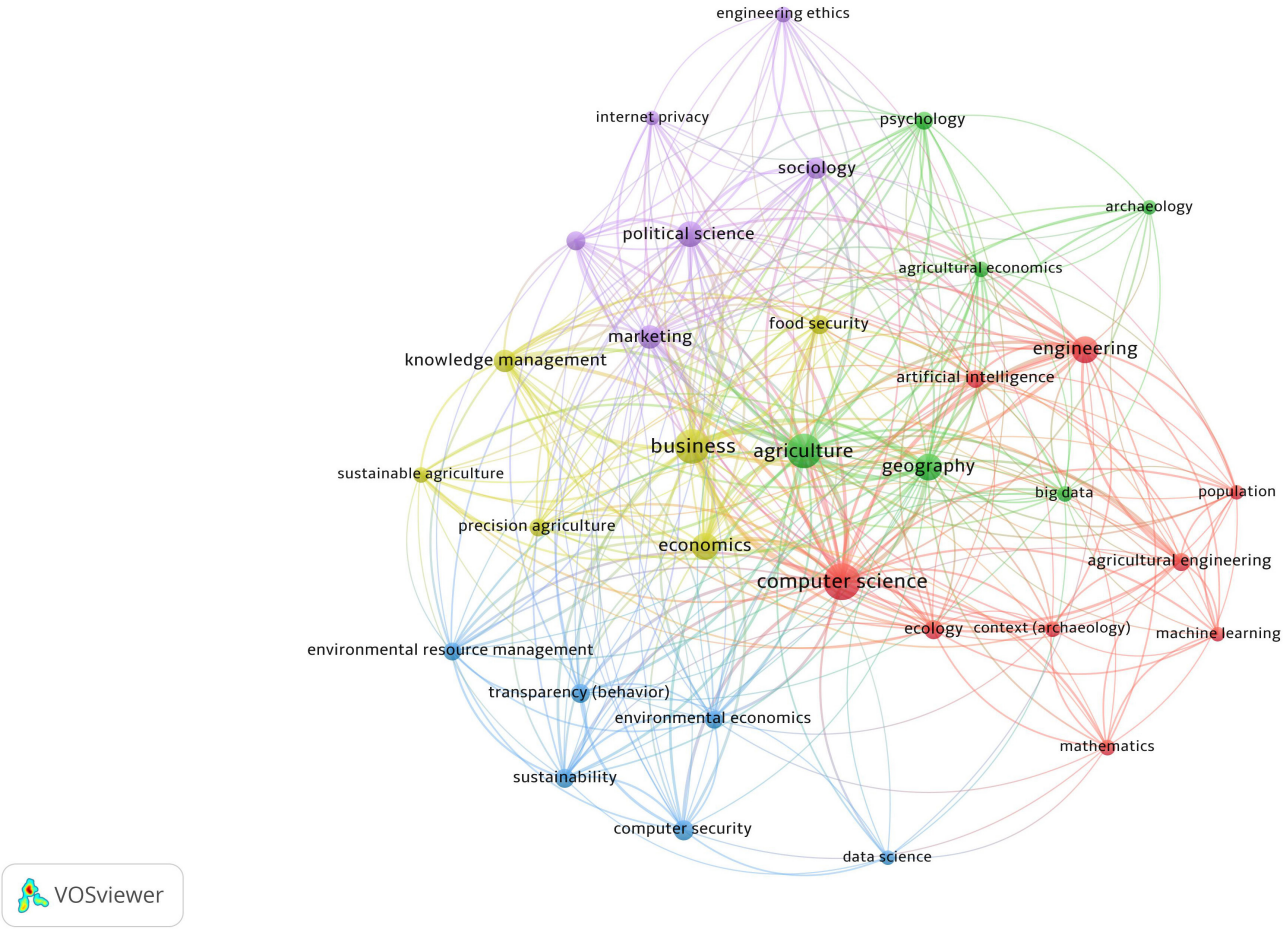
Keyword co-occurrence network of the reviewed literature generated using VOSviewer. Node size represents the frequency of keyword occurrence, and link strength indicates co-occurrence relationships between keywords. Colors represent thematic clusters identified by the clustering algorithm.

To better understand the thematic structure of the reviewed literature, a keyword co-occurrence network diagram was generated using VOSviewer (Van Eck and Waltman, 2010). Author keywords from the 38 selected papers were analyzed to identify frequently occurring terms and their relationships. Keywords appearing at least three times were included in the analysis to reduce noise. The resulting network diagram visualizes thematic clusters representing major research areas within the literature (Figure 4). The keyword network diagram reveals several interconnected clusters representing the multidisciplinary nature of AI adoption in agriculture. One cluster emphasizes technical aspects of artificial intelligence and machine learning methods, while another focuses on agricultural applications such as precision agriculture and sustainability. Additional clusters highlight socio-economic and governance dimensions, including knowledge management, institutional factors, and data governance. These clusters demonstrate that research on AI adoption in agriculture is spread across both technological development and socio-institutional considerations.

### 3.2 Analysis of results along the three dimensions

#### 3.2.1 Organizational scale

Fourteen of the reviewed papers address farming operations across all organizational scales (including small-scale, large-scale, and the intermediate scale). Twelve papers focus on small-scale farming operations, 8 discuss the intermediate scale, and 5 papers discuss large-scale farming operations. The remaining 5 papers provide no information regarding the organizational scale. The latter uses experimental or simulated datasets, with no real-world counterpart, to validate the AI-based agricultural system or model.

#### 3.2.2 Trust in AI/ML technology

Regarding trust in AI/ML technology, we find that 25 of the 38 analyzed papers mention farmers' hesitation (low trust) in employing AI/ML technologies for agricultural practices. A medium level of trust is mentioned in 7 of the analyzed papers, often describing mixed or context-dependent attitudes toward AI/ML technology adoption. For example, Mabkhot and Iqbal (2024) mention that farmers showed conditional trust in AI, influenced by social norms, peer approval, and community encouragement, rather than intrinsically motivated confidence in AI. Farmers showed a medium level of trust because they trusted the tools and advice from scientific experts, but feared underlying opaque data governance and inadequate protection (Hirunyatrakul, 2025; Yang et al., 2025). Marinko et al. (2023) state that farmers had low to medium trust in AI and ML technologies. Gardezi and Stock (2021) discuss the strategies agricultural technology companies use to foster high moralistic trust (Uslaner, 2002) in farmers to use AI and ML technologies. They propose the idea of moralistic trust, which means that farmers highly trust recommendations provided by AI/ML technology, not by carefully assessing risks and benefits, but through cultural transmission of idealized values of what it means to be a ‘good farmer' (Gardezi and Stock, 2021). Dilleen et al. (2023) discuss farmers' high trust in recommendations and advice given by peer farmers, farm advisors, and farming associations, but medium to low trust in recommendations from agribusiness and technology vendors. The remaining 5 analyzed papers provide no information about trust; however, these papers either mention that farmers in the respective study regions were unaware of high-tech AI/ML solutions (Dharmaraj and Vijayanand, 2018; Wolanin et al., 2020) or focus on technical or governance strategies that could foster farmers' trust in AI and ML technologies (Sabrina et al., 2022; Vardhan et al., 2024; Tahir et al., 2025).

#### 3.2.3 Digital divide

Twenty nine out of the 38 analyzed papers mention the digital divide in their text, with 25 papers emphasizing a high digital divide and 4 papers mentioning a medium digital divide. None of the papers mention a low digital divide; while 9 of the manuscripts do not address the topic of a digital divide. Among those that discuss the digital divide, not all provide detailed explanations or describe the reasons for the divide. Common themes include a lack of digital infrastructure in rural areas (Ryan et al., 2023; Jakku et al., 2019; Fleming et al., 2018; Deji et al., 2024), affordability constraints (Mabkhot and Iqbal, 2024; Tahir et al., 2025; Sparrow et al., 2021; Eastwood et al., 2019), and the fact that most AI/ML tools are designed for data-rich, well-connected, and often large–scale farms (Van Hilten et al., 2025; Hirunyatrakul, 2025; Dilleen et al., 2023; Gardezi et al., 2022; Hüllmann et al., 2023; Mallinger and Baeza-Yates, 2024).

**Figure 5:**
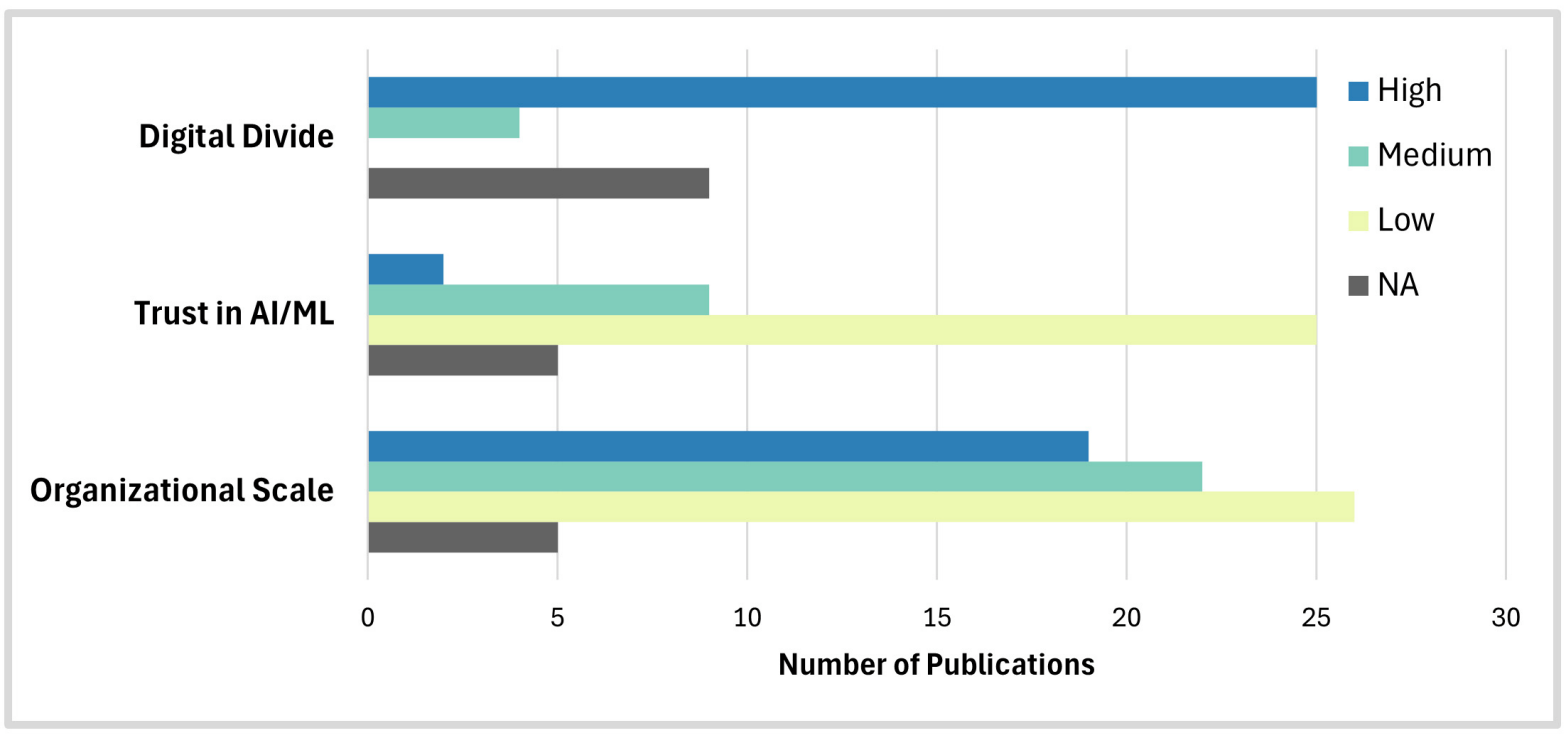
Summary of the number of publications across the three axes of the analysis framework. Some publications covered more than one organizational scale category.

Table 1 lists all manuscripts included in the analysis and their categorization along the three dimensions. While most manuscripts provide information on the organizational scale, trust, and digital divide, a few do not (five, five, and nine manuscripts, respectively). Furthermore, two manuscripts do not report information on any of the three dimensions. Figure 5 provides a summary of the distribution of categories across the three dimensions. The reviewed studies for this analysis include farms and farming operations of all scales, including large-, medium-, and small-scale, with most publications covering the small scale. Moreover, most publications report low trust in AI/ML technologies and a high digital divide (Figure 5). Of the three dimensions analyzed, the least discussed in the reviewed publications is the digital divide.

### 3.3 Challenges for trustworthy AI/ML technology in agriculture

**Figure 6:**
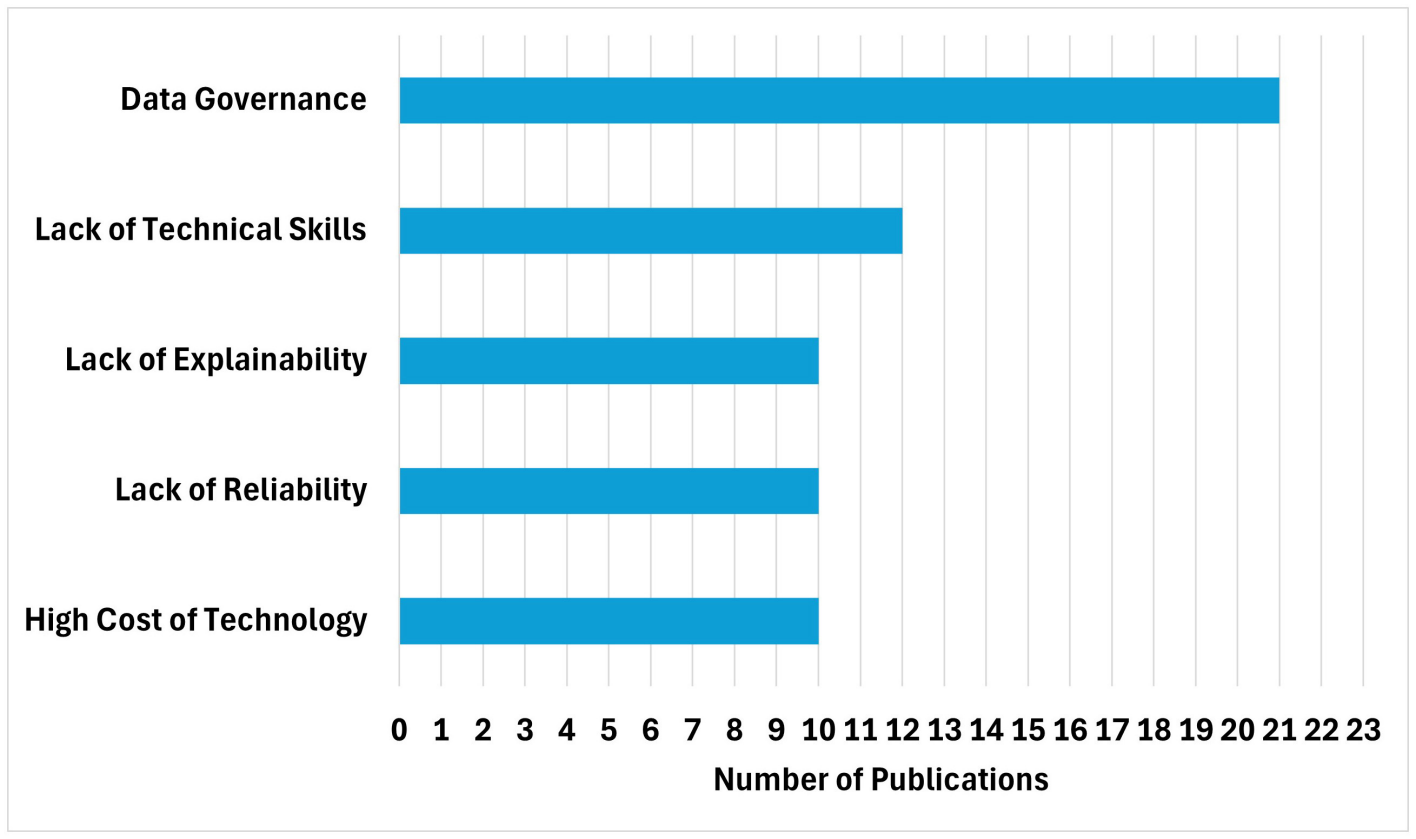
Number of publications (out of 38 total) that address each of the five thematic clusters of challenges for trustworthy AI/ML.

We find that despite significant advancements in AI and ML technology in recent years, which have led to vast opportunities in agricultural applications, its adoption has been relatively slow (Adereti et al., 2023; Dharmaraj and Vijayanand, 2018; Ryan et al., 2023). The analysis along the three dimensions reveals low trust in AI/ML technology and a high digital divide. To identify central challenges for trustworthy AI/ML in agriculture discussed across the included studies, a qualitative content analysis approach was employed. During full-text review, reported challenges, barriers, limitations, and concerns related to AI/ML adoption were extracted and recorded. Similar issues described across studies were grouped into higher-order categories through iterative comparison, yielding five thematic clusters. Individual studies contributed to multiple themes; therefore, the frequency of each theme was calculated based on the number of studies referencing the respective challenge. Data governance concerns are identified in 21 of the 38 reviewed manuscripts, making it the most frequently discussed challenge. Lack of technical skills and limited digital literacy among farmers are the second-most frequently mentioned themes, appearing in 12 of the reviewed manuscripts. The remaining three clusters, lack of explainability, lack of reliability, and high cost of AI/ML technology, are discussed in 10 reviewed manuscripts (Figure 6). Together, these themes or challenges illustrate how technical design choices, skills gaps, and economic and governance structures jointly shape farmers' ability and willingness to trust and use AI/ML in agriculture.

In the following, we discuss these five challenges related to trust facing farmers or their perception, potentially slowing their adoption of AI and ML technologies, namely (1) data governance, (2) lack of technical skills, (3) lack of explainability, (4) lack of reliability, and (5) cost of technology.

#### 3.3.1 Data governance

Data governance is the most frequently discussed challenge (Figure 6). The reviewed manuscripts report a variety of farmer concerns related to data privacy, security, ownership, access, sharing, licensing agreements, and benefit sharing (Van Hilten et al., 2025; Jakku et al., 2019; Alexander et al., 2024; Fleming et al., 2018; Sparrow et al., 2021; Eastwood et al., 2019; Adereti et al., 2023; Wiseman et al., 2019; Raturi et al., 2022; Rozenstein et al., 2024; Kramarz and Runowski, 2025). Several studies report that farmers were uncertain about how operational farm data (crop yields, fertilizer and pesticide management data, location-specific characteristics of soil, water, and micro-climate information, etc.) and personal data (financial records, insurance information, etc.) would be used, shared, and controlled by agribusinesses, technology providers, governments, and corporations (Dilleen et al., 2023; Fleming et al., 2018; Gardezi et al., 2022; Raturi et al., 2022; Rozenstein et al., 2024; Ryan et al., 2023; Wiseman et al., 2019). These concerns are related to personal data, such as personal financial records, geolocation data (Rozenstein et al., 2024), and also to sensitive information about farm management practices, preferences, and decision-making processes on the agricultural business and operation side (Gardezi et al., 2022), which farmers fear could be repurposed without their consent.

Across the data-governance cluster, recurring issues include concerns related to uncertainty over data ownership and control, opaque data access and licensing arrangements, and questions about which entity gains the financial benefits from data sharing (Raturi et al., 2022; Sparrow et al., 2021; Wiseman et al., 2019). For example, Sparrow et al. (2021) and Ryan et al. (2023) describe farmers' fears that AI-enabled systems might be used for surveillance of farm activities, and that sensitive data might be used or sold to other entities (such as regulators or commercial actors) to secure a negotiation advantage in the context of commercial arrangements and other economic purposes. Hirunyatrakul (2025) similarly report that Thai smallholders were concerned about opaque contractual terms and the possibility that platform providers might monetize detailed farm and personal data. In another example, Rozenstein et al. (2024) indicate concerns that governmental entities may use high-resolution agricultural data to monitor, control farmers, and impose additional regulations. Fleming et al. (2018) and Raturi et al. (2022) report that many farmers were reluctant to engage with AI-enabled platforms because they did not know which data were being collected, how long the data would be stored, or who would have access to the derived insight. Lack of clear communication and inaccessible terms of service further contribute to uncertainties surrounding data use (Jakku et al., 2019; Wiseman et al., 2019). Kramarz and Runowski (2025) mention that Polish farmers lacked trust in data-driven tools due to unclear data practices and limited ability to influence agreements.

The lack of transparency and clarity regarding data control and ownership shows a power imbalance between farmers and farming operations on the one hand and AI-/ML-technology companies and service providers on the other hand. (Jakku et al., 2019, 7) quote an Australian local government personnel stating: “…most farmers are still reasonably small, and most businesses they deal with are reasonably large, so there's going to be an inequity in the data”. Eastwood et al. (2019) identify imbalances in power relationships between farmers and private companies in New Zealand, centered on data collection and use. Data ownership is uncertain, and (Eastwood et al., 2019, 758) state that “this issue arose because some companies saw data as an asset to capture and control”. Making a similar argument, (Jakku et al., 2019, 6) state that “…many large corporations seem to be caught up in the idea that data will be valuable and want to own it and extract insights from it, possibly at the expense of local growers.” These examples emphasize farmers' concern that AI/ML technology or service providers may sell acquired data to third parties without consent (Ryan et al., 2023), potentially causing an economic disadvantage to farmers and farming operations (Mallinger and Baeza-Yates, 2024) and creating the potential for data misuse (Jakku et al., 2019). Additional data governance-related concerns include cybersecurity risks, with several papers noting that farmers feared hacking, unauthorized access, or data breaches in AI-enabled systems (Yang et al., 2025; Vardhan et al., 2024; Sparrow et al., 2021). In contexts involving smallholder farmers or “AI for social good” deployments, studies report uncertainty about data use in systems where farmers received free or subsidized access, but data exploitation occurred elsewhere in the value chain (Hirunyatrakul, 2025; Heldreth et al., 2021).

A consistent pattern across the reviewed papers is that data-governance concerns vary in type and intensity depending on the organizational scale of farming operations. Large-scale farms tend to generate extensive, high-resolution datasets through advanced sensors, machinery, and management systems (Sparrow et al., 2021). As a result, they encounter governance challenges related to data storage, platform dependence, and interoperability, often expressing concern about how governments or industry partners may use their data for compliance monitoring or market control (Sparrow et al., 2021; Eastwood et al., 2019; Rozenstein et al., 2024). In contrast, small-scale farms often lack bargaining power, legal literacy, and technical capacity to negotiate data-sharing terms, leading to fears of loss of ownership, unfair benefit distribution, and exploitation by technology vendors (Hirunyatrakul, 2025; Gardezi et al., 2022; Kramarz and Runowski, 2025).

Several reviewed papers emphasize that, because most AI/ML systems are developed and trained using data from large, well-digitized farms, small-scale farms are at a disadvantage (Van Hilten et al., 2025; Jakku et al., 2019; Eastwood et al., 2019; Gardezi et al., 2022; Mallinger and Baeza-Yates, 2024). Small-scale farming operations generate less data, have limited leverage in negotiating data-sharing terms, and often lack the infrastructure to benefit from the insights produced. This means that large farms help shape the design and performance of AI tools, while small farms face greater exposure to exploitation, misinterpretation, or loss of control over their data. Data governance challenges are not just technical problems but structural issues shaped by the scale of farming operations, economic power, and unequal access to digital resources (Jakku et al., 2019; Mallinger and Baeza-Yates, 2024; Raturi et al., 2022). These findings can be situated within the broader political economy of agri-food digitalization, where increasing concentration of platform ownership and limited farm-specific data governance frameworks reinforce power asymmetries between farmers and technology providers. From a governance perspective, this aligns with frameworks such as Responsible Research and Innovation (RRI), which emphasize transparency, inclusivity, and accountability in digital agriculture (Eastwood et al., 2019).

#### 3.3.2 Lack of technical skills

The reviewed manuscripts frequently report that the integration of AI/ML technology into agricultural practices introduces new skills and, hence, training requirements that many farmers and service providers find challenging to meet (Gardezi et al., 2022; Orn et al., 2020; Sparrow et al., 2021). Several studies note that farmers increasingly need to work with digital interfaces, sensors, data dashboards, and automated systems, in addition to their traditional agronomic skills. For example, Gardezi et al. (2022), report that to be successful, a farmer should be an observational data collector and skilled in using computers and technologies (including drones and tractors with GPS). Similarly, Sparrow et al. (2021) mention that AI-enabled systems will change farmers' jobs from manual labor-intensive to more white-collar, professional, a transformation already being promoted in advertisements from fertilizer, pesticide, seed, and farm machinery manufacturers.

However, multiple studies highlight skill gaps related to operating advanced technologies, interpreting model outputs, and integrating digital tools into existing workflows. Eastwood et al. (2019) note that there is a challenge in building farmers' and service providers' skills to use technology and data appropriately due to limited investments in technical staff training. Hüllmann et al. (2023) report that both agricultural workers and equipment manufacturers lack statistical and computational skills and are still figuring out what to do with the data they collect. According to Marinko et al. (2023), farmers lack trust in AI and ML technology and are hesitant to employ the technology because they lack exposure to and personal experience with the technology, and perceive a lack of technical skills required. Heldreth et al. (2021) and Czibere et al. (2023) report that farmers' limited literacy, exposure to technology, and multilingual needs create additional barriers to understanding and effectively using AI-enabled tools. These findings suggest that the challenge is not only a matter of technical skills, but also whether AI systems are designed to recognize and accommodate farmers' experiential and context-specific knowledge.

Several studies also emphasize generational differences in digital readiness. Farmers, especially older ones, are hesitant to adapt to this rapid change (Gardezi et al., 2022; Kramarz and Runowski, 2025). Gardezi et al. (2022, p. 233) state that the “…average age of a farmer in the US is 60 years, and most of them are hesitant about how to adapt to rapidly changing farm work”. Kramarz and Runowski (2025) find that lack of knowledge and training is one of the top barriers to adoption of AI/ML tools for agriculture among smallholder farmers in Poland, with many over 50 stating that they did not feel confident using digital tools or AI-enabled decision systems.

Several manuscripts describe training and educational interventions as potential ways to address these skill gaps. Orn et al. (2020) suggest that educating farmers about AI, its benefits, how it works, and how to use AI technologies can increase trust and motivate them to adopt AI/ML technologies in their farming practices. Bampasidou et al. (2024) emphasize the importance of agricultural extension, training, and higher-education programs for building digital literacy, data management skills, and AI competencies for future farmers and advisors. Marinko et al. (2023, p. 12) support this idea by stating that “…farmers already using the decision support systems, and/or attending demonstrations had a higher level of trust”.

The technical skill gaps identified in the reviewed studies connect with the digital divide, which shapes who can realistically participate in AI-enabled agriculture. Farmers with limited internet connectivity, outdated devices, or unreliable access to digital infrastructure face greater barriers to learning and using AI/ML tools, regardless of their willingness to adopt them (Alexander et al., 2024; Deji et al., 2024; Eastwood et al., 2019; Kramarz and Runowski, 2025). Several papers note that farms in underserved regions lack internet connectivity or encounter frequent disruptions, making it difficult to run cloud-based dashboards, update software, and access online training resources (Sparrow et al., 2021; Eastwood et al., 2019; Heldreth et al., 2021). Farmers unfamiliar with digital tools have fewer opportunities to build skills, whereas farmers already familiar with digital tools have access to updated hardware and stronger connectivity. This means they can use AI tools more consistently and build advanced skills, further widening the digital divide (Van Hilten et al., 2025; Sparrow et al., 2021; Czibere et al., 2023).

#### 3.3.3 Lack of explainability

A major challenge for building trust in AI and ML technology is the lack of explainability of how results are generated (Cartolano et al., 2022; Hüllmann et al., 2023; Sparrow et al., 2021). Here, explainability means that decisions, predictions, and recommendations made by AI/ML systems are justifiable with easy-to-understand and meaningful explanations (Hirunyatrakul, 2025; Cartolano et al., 2022). Explainability significantly increases farmers' trust in AI and ML technologies, thereby making them more effective in agricultural practices (Hüllmann et al., 2023). (Cartolano et al. 2022, p. 88) support this idea, stating that “… if users cannot understand why a model behaves as it does, they will tend not to use it.” Especially if AI/ML-derived recommendations deviate from typical or expected approaches, this creates uncertainty and skepticism. According to (Alexander et al. 2024, p. 167), “… growers often come from older, traditional cultures and communities and usually trust their gut feelings, their experiences, and historical data. […] [t]he unique nature of each farm and region, long years of experience, hard-earned knowledge, and intuitive methods guide decision making.” Therefore, even when a model generates a recommendation based on good-quality data and suggests improved decision-making, a farmer might hesitate to implement the recommendation if it does not align with the farmer's experience, and it is unclear how the recommendation was reached (Sabrina et al., 2022). This example highlights that trust is shaped not only by model transparency but also by the extent to which AI systems align with and incorporate farmers' existing knowledge and decision-making practices. Moreover, the farmer may not benefit (or learn) from the recommendation for future decision-making. Van Hilten et al. (2025) similarly report that stakeholders across the agri-food system want clear, domain-specific explanations for model behavior as a minimum requirement to trust AI. Tsakiridis et al. (2020, p. 185) state, “… by enabling users to visualize the inner manifestations of the rule-based system, the model is transparent and thus trustworthy.” Ryo (2022) and Wolanin et al. (2020) recommend various explainable AI and interpretable ML methods and models to enhance trust and explainability.

The lack of explainability is directly linked to farmers' overall trust in AI/ML systems. Across the reviewed manuscripts, trust was consistently lower when farmers could not easily understand how predictions were generated, which data sources were used, or why recommendations differed from their expectations or experience. Marinko et al. (2023) and Dilleen et al. (2023) show that farmers tend to trust advice from peers, agronomists, and extension agents far more than algorithmic outputs, because advisors can justify their reasoning, answer follow-up questions, and contextualize recommendations. Van Hilten et al. (2025) and Hirunyatrakul (2025) further emphasize that opaque or black-box AI systems undermine trust, particularly in small-holder and resource-constrained settings, because the perceived risk of making an incorrect decision is high and farmers heavily rely on their own experience and knowledge. Alexander et al. (2024) and Wolanin et al. (2020) report that trust decreases when models lack explainability; in such situations, farmers rely on their traditional practices and intuition.

#### 3.3.4 Lack of reliability

Several reviewed papers identify a lack of reliability in AI/ML technologies as significantly affecting farmers' trust in employing this technology (Alexander et al., 2024; Yang et al., 2025; Mallinger and Baeza-Yates, 2024; Heldreth et al., 2021). Agricultural decisions are critical, and the consequences may sometimes be irreversible, requiring them to be precise and dependable (Vardhan et al., 2024). For example, farmers may lose trust in the model's recommendations if it recommends over- or under-irrigation due to inaccuracy, and the resulting irrigation approach leads to crop damage. Heldreth et al. (2021) find that insufficient fertilizer recommendations and unreliable weather predictions lead to distrust in AI technology and skepticism about its benefits. Gardezi et al. (2022) mention that uncertainty in recommendations made by AI/ML technology stems from the inability of a model to accurately account for the variability found in natural systems. This concern is reinforced by Hüllmann et al. (2023), who find that model performance varied widely across fields and seasons, leading users to question the robustness of predictions in real-world conditions. Kramarz and Runowski (2025) mention that farmers perceived AI-generated advice as unreliable when predictions conflicted with observable field conditions or failed to incorporate localized agronomic knowledge.

Mallinger and Baeza-Yates (2024) highlight that incomplete or low-quality datasets used to train agricultural AI models contribute to inconsistent predictions, particularly in heterogeneous farming environments, resulting in a lack of reliability. This problem is further exacerbated by the lack of validation for AI/ML technology. Jakku et al. (2019, p. 6) state that “…being a relatively new field, it is going to take some time to get validation and to get the systems working at a high level of accuracy.” The lack of reliability directly affects farmers' trust in AI/ML systems and often becomes a barrier to adoption. Across the reviewed manuscripts, authors mention that trust declined when model outputs were inconsistent, contradicted field conditions, or did not align with farmers' experience and knowledge. Without consistent, validated performance, farmers perceive traditional knowledge and observational skills as safer and more dependable (Marinko et al., 2023; Dilleen et al., 2023; Rose et al., 2021).

#### 3.3.5 High cost of technology

Incorporating AI and ML technologies into agricultural practices requires high initial investments, significant new changes in the existing system, and high maintenance costs without the certainty of return or profit (Gardezi et al., 2022; Rozenstein et al., 2024; Tsakiridis et al., 2020). (Gardezi et al. 2022, p. 236) quote a small-holder farmer: “…it is challenging to get a return on investment on some of these precision agriculture technologies if you don't have enough acres or volume to spread it over”. Furthermore, (Hüllmann et al. 2023) echo that farmers consider their farms a business, and although they appreciate the benefits of using AI systems, they lack trust in the profitability of these systems. (Rozenstein et al. 2024) point out that economic value depends on the decisions that change in response to access to recommendations from an AI system, but it is often difficult to know whether those changes actually increased profitability compared to decisions made without AI/ML systems. Similarly, (Kramarz and Runowski 2025) mention that smallholder farmers “could not justify” high investments in equipment and software given their limited profitability. Similarly, (Mallinger and Baeza-Yates 2024) and (Bampasidou et al. 2024) report that many AI systems are designed around datasets and operational assumptions of large-scale industrial farming. These systems often perform inconsistently in heterogeneous or resource-constrained environments, further increasing farmers' hesitation to invest.

Several studies highlight the cumulative nature of AI-related costs. (Hüllmann et al. 2023) report that even when farmers appreciate the potential efficiencies of AI systems, they doubt the long-term profitability once training, maintenance, subscription fees, data-storage costs, and required hardware upgrades are factored in. For smallholder farmers in the Global South, Heldreth et al. (2021) and Gent (2025) emphasize that economic uncertainty amplifies these concerns. For example, many farmers rely on shared or low-end digital devices, lack access to credit, or cannot afford the recurring fees associated with proprietary AI-based decision-support systems. Even when AI tools promise financial gains through optimized irrigation, reduced input use, or improved pest forecasting, benefits may outweigh the financial costs of adopting and integrating new systems (Alexander et al., 2024; Adereti et al., 2023; Gent, 2025).

The cost of AI/ML technologies is strongly linked to the organizational scale of a farming operation. Larger, more capital-intensive farms are better positioned for high upfront investments, maintenance fees, and subscription costs, making AI/ML adoption economically viable (Gardezi et al., 2022; Hüllmann et al., 2023; Kramarz and Runowski, 2025). In contrast, small and medium-scale farms operate with tighter margins and limited access to financing. Several reviewed papers note that AI/ML tools are designed and tested in large, data-rich farming operations (Mallinger and Baeza-Yates, 2024; Bampasidou et al., 2024). When smaller farms adopt these tools, expected returns may be lower or more uncertain because fixed costs cannot be spread over a large area, making management costs per unit area higher than on large farms.

## 4 Discussion

### 4.1 Dimensions of the analysis framework

#### 4.1.1 Organizational scale

The organizational scale controls both access to AI/ML technologies and the degree of trust farmers place in them. Most of the reviewed manuscripts (see Supplementary material S2) indicate that AI and ML technologies favor larger-scale farming operations. These farming operations have greater value chain integration and access to higher-quality information. These factors allow them to benefit more than small-scale farming operations, which tend to have limited access to information and are more risk-prone (Fleming et al., 2018). In addition, the development of ML models favors large-scale farms over small-scale farms, making the resulting models a better fit for larger-scale operations (Gardezi et al., 2022). This often increases their feasibility for using digital systems and their potential benefits. At the same time, smallholders and medium-scale farms face steeper per-hectare costs and weaker bargaining positions in data and technology contracts (Hirunyatrakul, 2025; Mallinger and Baeza-Yates, 2024; Rozenstein et al., 2024; Kramarz and Runowski, 2025).

Yet willingness to adopt AI/ML technology is not confined to large-scale farming operations. Several studies show strong interest among smallholders when solutions demonstrably reduce risk or increase yield, especially under conditions of economic uncertainty (Hirunyatrakul, 2025; Kramarz and Runowski, 2025; Heldreth et al., 2021; Gent, 2025). Farmers at all scales seek to adopt AI/ML technologies in their agricultural practices to remain competitive in the agricultural sector (Orn et al., 2020). Farmers consider cost savings, particularly on seeds and fertilizers, and labor savings, among the benefits of employing AI technologies in their farming practices (Dilleen et al., 2023). These efficiency gains are especially appealing to medium and large-scale farms, where operational scale can increase the economic benefits of optimized input allocation. Yet, smallholders also express interest when tools address their specific constraints. Kramarz and Runowski (2025) find that smallholder farmers in highly fragmented landscapes expressed interest in digital tools when they believed such tools could help them manage dispersed plots more efficiently or identify opportunities for incremental productivity gains. Gent (2025) show that Indian farmers were motivated to adopt AI-based advisory services when these tools produced observable improvements in water-use efficiency and pest management, even if trust in the underlying algorithms remained conditional. Similarly, Hirunyatrakul (2025) report that Thai smallholders adopted AI tools when they were offered immediate economic relief, such as higher yields, improved credit access, or better market prices, emphasizing the role of functional trust based on perceived benefits in motivating adoption.

#### 4.1.2 Trust

Our analysis identified two different types of trust: trust regarding the use of personal and farming data and trust regarding the performance of AI/ML technology. Trust (or the lack thereof) in personal and farming data stems from farmers‘ concern about what data is collected, how it is used, and what control they will have over it. Farmers are facing a dilemma because they want to own and have control over their sensitive data, but also want to provide their data to benefit from AI/ML systems. Raturi et al. (2022) suggest that ensuring transparency and accountability in managing and sharing data and clearly defining which data should be kept confidential and which data should be shared more widely, would help increase farmers' trust. Lack of trust in the performance of applied AI/ML systems arises when the AI/ML technology is unreliable, and the recommendations it generates are not explainable. The two types of trust are interconnected; reliable models are required to build trust, and to make reliable models, data is required, but farmers are skeptical about data sharing. Also, the livelihoods of farmers may depend on the performance of the technology, so farmers have to trust a good and reliable outcome that justifies the resource investment (Gardezi et al., 2023). This also means that trust in AI/ML technology among farmers will not be obtained unless technical performance, governance, and social meaning are jointly addressed. However, findings from several reviewed studies suggest that these two forms of trust may not develop simultaneously. In some cases, farmers adopt or continue using AI/ML technologies when performance benefits such as improved yields, cost savings, or risk reduction are clearly demonstrated even if concerns about data governance remain unresolved (Hirunyatrakul, 2025; Gent, 2025). Thus, performance trust can emerge independently of, and sometimes prior to data trust. Furthermore, trust-building in AI may be sequential, where early adoption is driven by observable benefits, while sustained and broader trust depends on addressing longer-term concerns related to data ownership, control, and governance.

Low trust is present in smallholder systems in the Global South, family farms in Europe and North America, and among large-scale commercial operations (Ryan et al., 2023; Van Hilten et al., 2025; Gardezi et al., 2022; Hüllmann et al., 2023; Wiseman et al., 2019; Kramarz and Runowski, 2025; Heldreth et al., 2021; Orn et al., 2020). Some of the reviewed manuscripts discuss the different types of concerns for small-scale and large-scale farms that lead to low trust in AI/ML technologies. For example, even though both large-scale and small-scale farming operations have low trust levels due to data governance concerns, large-scale farmers are more concerned about the data storage processes and uncertainty in government regulations and changes thereof, while small-scale operations are more concerned about maintaining the rights to their data and about benefiting from it (Fleming et al., 2018). Since farmers, regardless of the scale of their farming operations, consider themselves business owners aiming to make a profit, they are sensitive to the uncertainties and risks of using AI/ML technology, and hence, are skeptical about incorporating it into their agricultural activities and workflows. Our analysis suggests that farmers' skepticism toward AI/ML can be reduced if farmers are provided with recommendations and advice streamlined to be in line with their thought processes, the inner workings of a farming operation, and clear and transparent explanations of what, when, and how of which actions will be implemented (Alexander et al., 2024; Mallinger and Baeza-Yates, 2024; Sabrina et al., 2022). These findings highlight the importance of explainability for uptake of AI/ML technology.

#### 4.1.3 Digital divide

All reviewed manuscripts mentioning the digital divide identified this topic as one of the barriers to adopting AI/ML technology in agriculture. The main reasons cited for a high digital divide are limited internet access and the lack of proper digital infrastructure in rural areas (Fleming et al., 2018; Jakku et al., 2019; Marinko et al., 2023). Lack of knowledge about high-tech AI/ML solutions is also one of the reasons for the digital divide, especially in developing countries (Dharmaraj and Vijayanand, 2018). Another factor playing into the digital divide is that AI/ML technologies are often developed using a single framework designed for all types of farming operations. This means farming operations that align best with the data used for training AI/ML systems might benefit most from the new technology (Sparrow et al., 2021). Since large-scale farms generate more data and can adopt digital technologies at higher rates, most AI/ML models and tools work best for this category of farming operation, leading to a further widening of the digital divide (Mallinger and Baeza-Yates, 2024). This association creates a positive feedback loop in which AI systems, often developed using data from large, well-digitized farms, are better suited to those environments. As a result, these farms benefit more from AI adoption, while smaller or less digitized farms remain underrepresented, further reinforcing existing inequalities in access, performance, and trust. Also, large-scale monoculture farming operations are more straightforward to model and support than small-scale farming operations with diverse crop production (Gardezi et al., 2022). Our review also found that the greater the digital divide, the lower the trust. The more farmers know about specific technology, the more willing they are to try it.

### 4.2 Cross-dimensional interaction between scale, trust, and digital divide

**Figure 7:**
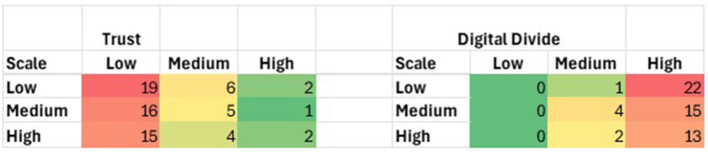
Cross-dimensional interaction between organizational scale, trust in AI/ML technologies, and the digital divide in the reviewed studies. The left panel shows the distribution of reviewed studies by organizational scale and reported trust levels in AI/ML technologies. The right panel shows the distribution of studies by organizational scale and digital divide conditions. Number in each cell represents the number of reviewed studies coded in each category.

To understand how the three analytical dimensions interact, the distribution of reviewed studies was examined across the combinations of organizational scale, trust in AI/ML technologies and the digital divide (Figure 7). While these dimensions were discussed individually in the earlier section, the cross-dimensional comparison reveals several patterns. Across all three scales, low trust in AI/ML technologies appears most frequently, indicating that skepticism toward AI systems is widespread in the agricultural literature regardless of farm size. However, the distribution varies slightly by scale. Small-scale farming contexts show the highest concentration of low-trust, followed by medium and large-scale operations, while medium and high trust levels occur only in a small number of studies. Similarly, across all three scales, high digital divide appears most frequently, with a variation by scale. Highest concentration of digital divide is seen in small-scale farm, followed by medium and large-scale operations. These patterns suggest that regardless of the organizational scale, there is low trust in AI/ML technologies and a high digital divide. However, it is important to note that the organizational scale classifications are derived from the definitions used in the original studies and are not globally standardized. As a result, categories may represent different structural and socio-economic conditions across regions. Therefore, these cross-scale comparisons should be interpreted as descriptive patterns within the literature rather than as directly comparable categories across geographic contexts.

### 4.3 Willingness to adopt and implement AI/ML

Despite substantial concerns about trust, skills, cost, and governance, our analysis reveals some straightforward but important reasons why farmers want to adopt and implement AI in their agricultural practices. The most frequently mentioned driver of adoption was the expectation that AI-enabled tools would improve efficiency and increase productivity, and profitability (Jakku et al., 2019; Alexander et al., 2024; Dilleen et al., 2023; Dharmaraj and Vijayanand, 2018; Tahir et al., 2025; Orn et al., 2020; Czibere et al., 2023; Durrant et al., 2021). Jakku et al. (2019) point out that farmers believe that data-driven AI technologies can increase efficiencies, productivity, and profitability by improving farm management and decision-making through targeted software applications. Also, linking data sets like soil, climate projections, weather forecasts, water models, and crop information can provide greater insights into addressing climate change-related challenges that ultimately benefit farmers (Cartolano et al., 2022; Jakku et al., 2019; Raturi et al., 2022).

Gardezi and Stock (2021) mention cultural reasons for farmers to adopt AI technologies (i.e., to become “successful” and “modern”). Mabkhot and Iqbal (2024) highlight that subjective norms, such as peer approval, institutional encouragement, and community-level support, strongly influence the willingness to adopt AI-enabled technologies. These findings show that farmers' willingness to adopt AI/ML stems from a combination of expected agronomic and financial benefits, improved resource efficiency, competitive pressures, and social or cultural motivations. Adoption is also driven by functional trust, confidence built through demonstrated and observable improvements in farming outcomes.

## 5 Conclusions

Our findings indicate that across diverse regions and production systems, AI/ML technology holds strong potential to improve efficiency, profitability, and resource management, yet widespread adoption remains constrained by structural, technical, and economic barriers. Our analysis identifies five interconnected challenge clusters, namely, data governance, lack of technical skills, lack of explainability, lack of reliability, and high cost of technology, that collectively limit trust in and uptake of AI/ML technology. These challenges are shaped by organizational scale. Large operations benefit from data richness, better connectivity, and lower per-unit costs, while intermediate and small-scale farms face higher financial risk, limited bargaining power, and reduced access to infrastructure. This reinforces an uneven technological landscape in which AI/ML performs best where it is already easiest to implement. A large share of global agriculture falls into the intermediate organizational scale, where current applications do not perform well enough to be trustworthy. This study highlights intermediate organizational scale as a critical but underrepresented target for AI/ML development. Hence, we recommend that future research and system design should prioritize solutions that are adaptable, cost-effective, and robust across farming contexts.

Our analysis indicates that farmers across all scales express a strong interest in adopting AI/ML technologies when tools demonstrably reduce risk, improve yields, or solve immediate operational challenges. The review also highlights the central role of transparency, data rights, and explainability in shaping farmers' perceptions. Ambiguity around who controls data, how information is shared, and how recommendations are generated continues to create hesitation, even among farmers willing to experiment with digital tools. Therefore, an additional recommendation to increase trust in AI/ML technology is to improve the transparency of developed systems by strengthening governance frameworks, clarifying benefit-sharing arrangements, and increasing the interpretability of model outputs. Increased education and training for farmers on AI/ML technology will also be essential to build trust and facilitate adaptation. It is likely to decrease the digital divide and considerably increase trust in new technology. Closing the geographic gap in AI/ML technology for agricultural applications in middle- and lower-income countries is a pressing need that can accelerate programs toward achieving the Zero Hunger SDG.

### 5.1 Limitations

This review has several limitations. First, it is restricted to English-language publications, which may exclude relevant research from other regions where English is not widely used for research publications. Second, the reviewed literature is concentrated in high-income contexts, which may limit the generalizability of findings to lower-income agricultural systems. Third, classifications of farm scale and trust are based on reviewed studies and are not standardized across contexts, which may affect cross-study comparability.

## Supplementary material

Supplementary Table S1 Search Query Used for various databases

Supplementary Table S2 Quality Assessment

Supplementary Table S3 PRISMA Checklist
