# Supplementary material for: Scale, trust, and the digital divide: a systematic review of AI and ML for agricultural applications
Source: Front Artif Intell. 2026 Jun 19;9:1798896. doi: 10.3389/frai.2026.1798896 (PMC13328088; doi:10.3389/frai.2026.1798896)
Supplement: Supplementary Table S1 — Search Query Used for various databases [file Table_1.docx]

**1. Search Query used for Google Scholar**

("AI" OR "artificial intelligence" OR “machine learning” OR “data science”) AND ("agri*" OR "farm*" OR “digital agriculture” OR “precision agriculture”) AND ("trustworthy" OR “trustable” OR “transparent” OR "reliable" OR "trust" OR "explainable” OR "XAI" OR "interpretable" OR "ethical")

**without the words** “review ", "systematic review", "meta-analysis" and "literature review"

**2. Search Query used for Web of Science**

TS = ("AI" OR "artificial intelligence" OR “machine learning” OR “data science”) AND

("agri*" OR "farm*" OR “digital agriculture” OR “precision agriculture”) AND

("trustworthy" OR “trustable” OR “transparent” OR "reliable" OR "trust" OR "explainable” OR "XAI" OR "interpretable" OR "ethical")

NOT TS = (“systematic review”)

NOT TS= (“remote sensing”)

**3. Search query used for IEEE Xplore**

(“All Metadata”: "AI"

OR “All Metadata”: "artificial intelligence" OR “All Metadata”: “machine learning”

OR “All Metadata”: “data science”)

AND

(“All Metadata”: "agri*" OR “All Metadata”: "farm*"

OR “All Metadata”: “digital agriculture” OR “All Metadata”: “precision agriculture”)

AND

(“All Metadata”: "trustworthy" OR “All Metadata”: “trustable”

OR “All Metadata”: “transparent” OR “All Metadata”: "reliable"

OR “All Metadata”: "trust" OR “All Metadata”: "explainable”

OR “All Metadata”: "XAI" OR “All Metadata”: "interpretable"

OR “All Metadata”: "ethical")

NOT (“All Metadata”: “systematic review”) NOT (“All Metadata”: “remote sensing)
